# Incidence of numerical variants and transitional lumbosacral vertebrae on whole-spine MRI

**DOI:** 10.1007/s13244-016-0468-7

**Published:** 2016-02-12

**Authors:** Bernhard J. Tins, Birender Balain

**Affiliations:** Department of Radiology, The Robert Jones and Agnes Hunt Orthopaedic Hospital NHS Trust, Twmpath Lane, Oswestry, Shropshire SY10 7AG UK; Department of Spinal Surgery, The Robert Jones and Agnes Hunt Orthopaedic Hospital NHS Trust, Oswestry, Shropshire UK

**Keywords:** MRI, Spine, Lumbar vertebrae, Incidence

## Abstract

**Objectives:**

This study sets out to prospectively investigate the incidence of transitional vertebrae and numerical variants of the spine.

**Materials and methods:**

Over a period of 28 months, MRIs of the whole spine were prospectively evaluated for the presence of transitional lumbosacral vertebrae and numerical variants of the spine.

**Results:**

MRI of the whole spine was evaluated in 420 patients, comprising 211 female and 209 male subjects. Two patients had more complex anomalies. Lumbosacral transitional vertebrae were seen in 12 patients: eight sacralised L5 (3 male, 5 female) and four lumbarised S1 (3 male, 1 female). The incidence of transitional vertebrae was approximately 3.3. % (14/418). Thirty-two (7.7 %) of 418 patients had numerical variants of mobile vertebrae of the spine without transitional vertebrae. The number of mobile vertebrae was increased by one in 18 patients (12 male, 6 female), and the number was decreased by one in 14 patients (4 male, 10 female).

**Conclusions:**

Numerical variants of the spine are common, and were found to be almost 2.5 times as frequent as transitional lumbosacral vertebrae in the study population. Only whole-spine imaging can identify numerical variants and the anatomical nature of transitional vertebrae. The tendency is toward an increased number of mobile vertebrae in men and a decreased number in women.

*Main messages*

*• Numerical variants of the spine are more common than transitional vertebrae.*

*• Spinal numerical variants can be reliably identified only with whole-spine imaging.*

*• Increased numbers of vertebrae are more common in men than women.*

*• Transitional lumbosacral vertebrae occurred in about 3.3 % of the study population.*

*• The incidence of numerical variants of the spine was about 7.7 %.*

## Introduction

Transitional lumbosacral vertebrae are a common finding on spinal imaging, with reported incidences of 1–30 %, depending on the definition and imaging modality used [1–8]. These spinal variants are generally easier to appreciate on radiographs or CT than on magnet resonance imaging (MRI) [1, 8]. Various landmarks have been proposed to identify the anatomical level of the transitional vertebra on MR imaging. For example, various vascular structures and the location of the iliolumbar ligament have been investigated for this purpose. Ultimately, however, all these landmarks have been shown to be unreliable [1, 9, 10].

A more insidious problem is the case of morphologically normal lumbar vertebrae but an abnormal number of mobile vertebrae of the spine. Counting from the top of the spine, there might be an increased or decreased number of morphologically normal mobile vertebrae. This may be impossible to appreciate on MR imaging of the lumbar spine alone. However, it is important to realise the presence of a numerical variant, as this will result in variation in neural anatomy and may lead to misattribution of clinical symptoms, i.e. clinical findings or symptoms related to an incorrect spinal level, which may lead to inappropriate intervention.

Several authors have suggested that the only reliable method for determining the anatomical level is to count down from the atlantoaxial joint. Obviously, this requires imaging of the entire spine [2, 5, 11].

Anecdotally, there is a lack of awareness amongst radiologists regarding anatomical variation, particularly with regard to the frequency of numerical variants of the spine in the absence of morphological transitional vertebrae.

This paper sets out to determine the incidence of numerical variants and transitional lumbosacral vertebrae of the spine in consecutive patients in routine MR imaging practice.

## Materials and methods

From 1 January 2013 to 30 April 2015, all MRIs of the whole spine presented to the main author were prospectively assessed for the presence of numerical variants of the spine and the presence of a morphological transitional lumbosacral vertebra.

The vertebrae were counted from the C2 vertebra downwards, assuming seven cervical and 12 thoracic vertebrae. Although variation in the number of cervical and thoracic vertebrae has been described, variation in the cervical spine is very uncommon [12]. In the thoracic spine, it can be difficult to determine the anatomical nature of vertebrae at the thoracolumbar junction. The generally accepted convention is to attribute seven vertebrae to the cervical and 12 vertebrae to the thoracic spine [10, 11, 13].

In this study, vertebrae were considered transitional based on the criteria described by O’Driscoll et al. [8]. The vertebra must show some features of both a lumbar and sacral vertebra: “a well-formed residual disc between S1 and the remainder of the sacrum but also with an abnormal sagittal outline to the sacrum, i.e. “squaring” of the presumed upper sacral segment” [8].

The spine was assessed solely on the basis of the MRI appearances and not in conjunction with other imaging modalities.

In the case of transitional lumbosacral vertebrae, their nature was determined by counting down from the top, assuming seven cervical and 12 thoracic vertebrae (please see above). In the case of the transitional vertebra, it was called sacralised L5 if it followed after L4, and it was called lumbarised S1 if it followed after L5.

The routine MRI protocol consisted of sagittal T1-weighted (T1W), sagittal short tau inversion recovery (STIR) and sagittally acquired 3D SPACE [sampling perfection with application-optimised contrasts using different flip-angle evolution] sequences. The 3D SPACE sequences were reformatted in the axial and coronal planes.

In select cases, additional sequences were performed.

The sagittal images were acquired in two blocks of 38 cm craniocaudal size. For the assessment of the number of vertebrae, the composed view was reviewed using the image composer of the MRI scanner (*syngo* MR B17; Siemens Healthcare GmbH, Erlangen, Germany).

All examinations were performed on a MAGNETOM Avanto 1.5T MRI scanner (Siemens Healthcare GmbH).

All MRIs of the whole spine were analysed, regardless of the clinical indication for the examination.

## Results

From 1 January 2013 to 30 April 2015, MRIs of the whole spine in 420 patients (211 female and 209 male) were performed and reviewed by the main author.

**Fig. 1:**
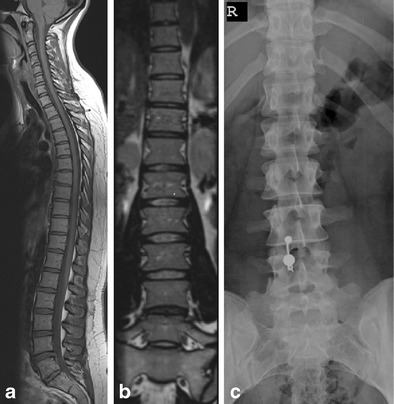
26-year-old woman with thoracic pain. MRI of the spine with sagittal T1-weighted images (**a**) and a coronal reformat of a sagittal SPACE sequence (**b**) demonstrates a transitional lumbosacral vertebra. Counting from the top, this is a sacralised L5 vertebra. The coronal reformats are very helpful in identifying the transitional nature of this vertebra. It is more easily appreciated on an anterior-posterior (AP) radiograph of the lumbar spine (**c**)

**Fig. 2:**
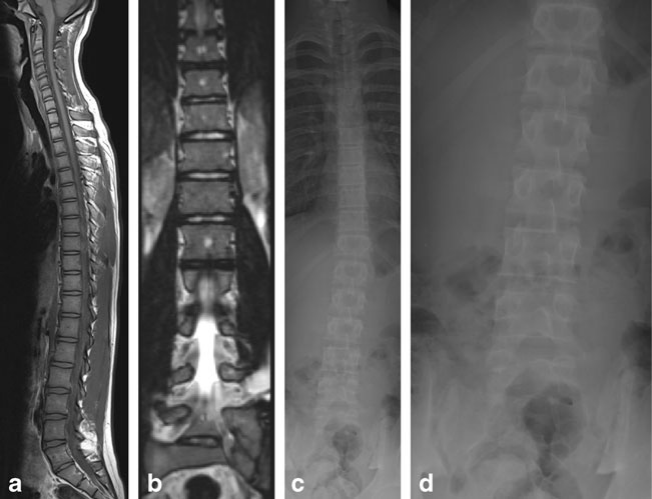
15-year-old boy with back pain. Sagittal T1-weighted image (**a**) and coronal reformats of a sagittal SPACE sequence (**b**) demonstrate a transitional lumbosacral vertebra. Counting from the top, this is a lumbarised S1 vertebra. This can also be identified on an anterior-posterior (AP) radiograph (**c**) of the whole spine, better appreciated on a magnified view of the lumbar spine only (**d**). The only reliable method for determining the nature of a transitional vertebra is to count from the top

Transitional lumbosacral vertebrae affecting either L5 or S1 were seen in 12 patients; in eight, L5 was partially sacralised (Fig. 1), and in four, S1 was partially lumbarised (Fig. 2). Among the eight patients with partial sacralisation of L5, three were male and five were female. The group of four with partial lumbarisation of S1 comprised three male and one female patient.

**Fig. 3:**
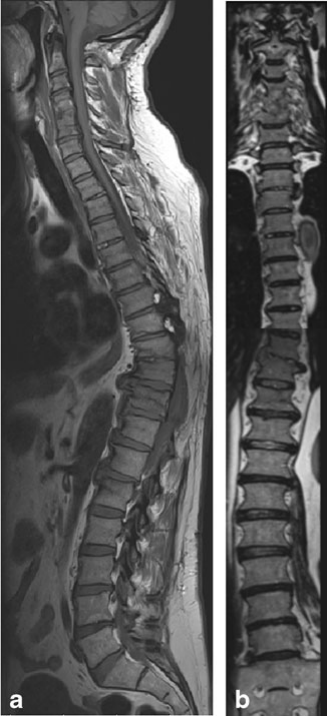
55-year-old man with spinal injury in the past. Sagittal T1-weighted images (**a**) and coronal reformats of a 3D SPACE sequence (**b**) demonstrate old injuries, fusion of cervical vertebrae C4 to C6 (most easily appreciated when counting the posterior elements), and one supernumerary vertebra. Additional mobile vertebrae are more common in men than in women

**Fig. 4:**
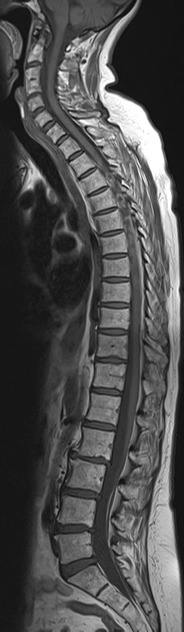
Sagittal T1-weighted MRI of the whole spine in a 63-year-old female patient with back pain. Block vertebra formation C3/4. The number of mobile vertebrae is reduced by one. Assuming seven cervical and 12 thoracic vertebrae, there are four morphological lumbar vertebrae, and there is no transitional vertebra as such

In 32 patients, the lumbosacral junction appeared morphologically normal on MRI but the number of vertebrae was abnormal, increasing by one in 18 patients (Fig. 3) and decreasing by one in 14 patients (Fig. 4). Among the 18 patients with an increased number of mobile vertebrae, 12 were male and six were female. Among patients with a decreased number of mobile vertebrae, four were male and ten were female.

Combining the number of patients with a reduced number of mobile vertebrae and with sacralisation of L5 results in a total of 22 patients, seven male and 15 female.

Combining the number of patients with an increased number of mobile vertebrae and with lumbarisation of S1 results in a total of 22 patients, 15 male and seven female.

This equates roughly to a ratio of 1/3 to 2/3.

In a further two patients (one male and one female), the number of free spinal vertebrae was increased by one, and there was also a transitional lumbosacral vertebra. Therefore, there were altogether 14 patients with transitional lumbosacral vertebrae.

In a further two patients (one male and one female), two balanced hemivertebrae were noted in the thoracic spine. These were in addition to a normal complement of mobile vertebrae, and might be seen as one additional vertebra. However, these two cases will be excluded from the further analysis, as the numbering of vertebrae is not straightforward, nor is it universally agreed upon in these circumstances. Also, the assumption of 12 thoracic vertebrae is no longer valid in these cases.

This results in an incidence of an increased or decreased number of vertebrae of 34/418, or 8.1 %. In 32 of 418 patients, or 7.7 % of cases, there was no morphological anomaly at the lumbosacral junction despite an anomalous number of mobile vertebrae.

As mentioned earlier, in two cases there were transitional lumbosacral vertebrae in addition to an increased number of mobile vertebrae.

Transitional lumbosacral vertebrae were seen in 14 of 418 or 3.3 % of patients.

There were four patients with known developmental anomalies. One was known to have Goldenhar syndrome, and this patient had one more mobile vertebra than normal and an additional transitional vertebra. One patient with known Langer-Giedion syndrome had one more mobile vertebra than normal. A patient with known Klippel–Feil syndrome and a patient with Arnold–Chiari malformation had a normal number of vertebrae.

The indications for the MRI examinations were quite varied, and included pain with suspected spinal origin, neurological symptoms with suspected spinal origin, inflammatory or infectious lesions of the spine, assessment of neoplastic disease of the spine, osteoporosis, spinal injury follow-up, and abnormal posture and alignment, including scoliosis.

## Discussion

The most striking finding of this study is the low number of morphological transitional lumbosacral vertebrae in the study population. Only 3.3 % of our patients demonstrated a transitional lumbosacral vertebra on MRI, using the criteria described by O’Driscoll et al. [8]. This is much lower than generally quoted in the literature. Only one study has noted a lower rate, at about 1 % [2], whereas many other studies have quoted the incidence of transitional lumbosacral vertebra in double digits, e.g. 10 % [7], 12 % [11], 15 % [8], 16 % [3], 18.6 % [1], and even up to 30 % [4].

The presence of a transitional lumbosacral vertebra does alert to the presence of anatomical variants in the neural anatomy and should trigger extra care when undertaking interventional procedures.

Anatomical variants without transitional morphology are more problematic because they can easily be missed. They were much more prevalent than morphologically transitional vertebrae in our study population, which showed a rate of 7.7 %. These patients will typically not be identified as having a numerical variant when only MR imaging of the lumbar spine is performed. Numerical variants of mobile vertebrae are associated with variation in the neural anatomy and can lead to inappropriate spinal intervention [3, 7, 14].

In men, there is a clear tendency toward an increased number of mobile vertebrae, either as true mobile vertebra or lumbarisation of a sacral vertebra, with a male-to-female ratio of 2:1.

The reverse is true in the case of a decreased number of mobile vertebrae or sacralisation of a lumbar vertebra, with a male-to-female ratio of roughly 1:2. The absolute combined numbers of increased or decreased mobile vertebra + transitional vertebra were very similar. However, for lumbarisation, there is a tendency to be complete—i.e. not transitional but fully mobile—while the opposite is true for sacralisation.

This study is the first to prospectively evaluate the presence of transitional lumbosacral vertebrae and or numerical variants on whole-spine MRI.

Hahn et al. [11] used a combination of scout views and MRI of the lumbar spine to look at transitional lumbosacral vertebrae, but did not differentiate between vertebrae with transitional morphology and numerical variants. The combined incidence in their study was 12 %, with a male predominance for lumbarisation of S1, similar to our study.

Hanson and coworkers [5] reviewed whole-spine MRI in 750 consecutive outpatients for the presence of numerical variants. Similar to Hahn et al., they did not separate vertebrae with transitional morphology from numerical variants. Assuming seven cervical and 12 thoracic vertebrae, they found a 20 % incidence of numerical variation, with 14.5 % of all patients having six lumbar vertebrae, 5.3 % having four, and one patient (0.13 %) having three. Eighty percent of patients in their study had five lumbar vertebrae. Interestingly, two-thirds of the patients with four lumbar vertebrae were female, while two-thirds of patients with six lumbar vertebrae were male, a trend also borne out in our study.

Akbar et al. [2] used a whole-spine localiser to assess numerical variants of the lumbosacral junction. They found overall incidence of 7.7 %, with only two of 207 patients (about 1 %) having transitional lumbosacral morphology on scout imaging. Therefore, the incidence of numerical variation in mobile vertebrae and the presence of transitional vertebrae in their study was even lower than in ours. However, this may have been due to limited quality of the scout images.

Coronal reformats of the 3D SPACE sequence were useful in identifying transitional vertebrae, as has been described elsewhere [15].

Intra- and interobserver variation was not assessed in this work. It stands to reason that there is some variability in the assessment of transitional vertebrae. While coronal reformats can provide some help, they are not always available, and radiographs remain the gold standard. The fundamental problem that remains, however, is that numerical variants are relatively common and cannot be appreciated unless whole-spine imaging is performed.

In our institution, the imaging protocol of MRI of the lumbar spine was modified in light of these findings. All patients now undergo imaging using a sagittal half-Fourier acquisition single-shot turbo spin-echo (HASTE) localiser of the spine in two sagittal blocks, which allows imaging of the whole spine in about 30 s, ensuring adequate determination of anatomical levels in all examinations.

## Conclusion

Numerical variants of the spine are common, and were almost 2.5 times as frequent as transitional lumbosacral vertebrae in the study population. While MRI of the lumbar spine can usually identify transitional lumbosacral vertebrae (though not as reliably as radiographs), it cannot identify numerical variants of the spine, which is possible only by imaging of the whole spine. In men, the tendency is toward an increased number of mobile vertebrae, whereas the number tends to decrease in women. An accurate determination of anatomical levels is a prerequisite for accurate assessment of pathological findings and for any image-guided spinal intervention.
